# Effects of low-dose tamoxifen on breast cancer biomarkers Ki-67, estrogen and progesterone receptors

**DOI:** 10.1186/1477-7800-3-29

**Published:** 2006-09-14

**Authors:** Juarez Antônio de Sousa, Gil Facina, Benedito Borges da Silva, Luiz Henrique Gebrim

**Affiliations:** 1 Division of Breast Diseases, HMI, Goiânia, Brazil; 2 Division of Breast Diseases of the Universidade Federal de São Paulo (UNIFESP), São Paulo, Brazil

## Abstract

Breast carcinoma is the most common malignancy among women and it has a major impact on mortality. Studies of primary chemoprevention with tamoxifen have generated high expectations and considerable success rates. The efficacy of lower doses of tamoxifen is similar to that seen with a standard dose of the drug, and there has been a reduction in healthcare costs and side effects.

The immune reaction to monoclonal antibody Ki-67 (MIB-1) and the expression of estrogen receptors (1D5) and progesterone receptors (PgR 636) in breast carcinoma were studied in patients treated with 10 mg of tamoxifen for a period of 14 days.

A prospective randomized clinical trial was conducted with 38 patients divided into two groups: Group A: N = 20 (control group-without medication) and Group B: N = 18 (tamoxifen/10 mg/day for 14 days). All patients signed an informed consent term previously approved by both institutions. Patients underwent incisional biopsy before treatment and 14 days later a tumor tissue sample was obtained during surgical treatment. Positivity was quantitatively assessed, counting at least 1.000 cells per slide. For statistical data analysis, a Wilcoxon non-parametric test was used, and α was set at 5%.

Both groups (A and B) were considered homogeneous regarding control variables. In Group A (control), there was no statistically significant reduction in Ki-67 (MIB-1) (p = 0.627), estrogen receptor (1D5) (p = 0.296) and progesterone receptor positivity (PgR 636) (p = 0.381).

In Group B (tamoxifen 10 mg/day), the mean percentage of nuclei stained by Ki-67 (MIB-1) was 24.69% before and 10.43% after tamoxifen treatment. Mean percentage of nuclei stained by estrogen receptor (1D5) was 59.53% before and 25.99% after tamoxifen treatment. Mean percentage of nuclei stained by progesterone receptor (PgR 636), was 59.34 before and 29.59% after tamoxifen treatment. A statistically significant reduction was found with the three markers (p < 0.001).

Tamoxifen significantly reduced monoclonal antibody Ki-67 (MIB-1), estrogen receptor (1D5) and progesterone receptor positivity (PgR 636) in the breast epithelium of carcinoma patients treated with a 10 mg dose of tamoxifen for 14 days.

## Background

Chemoprophylaxis of breast carcinoma with tamoxifen in women at increased risk for breast cancer has produced encouraging results. Studies of primary chemoprevention with tamoxifen in usual doses have generated high expectations with a 50% reduction in the relative risk for invasive carcinoma in high-risk patients [1]. Some evidence indicates that tamoxifen dose can be lowered to reduce its side-effects and retain drug efficacy [1-3].

Tamoxifen and similar drugs act in a complex manner as estrogen antagonists in tumor tissue, and as estrogen agonists in other body tissues, including the bones, uterus and cardiovascular system. The drug's action is mediated by cell estrogen receptor through various complex reactions [4-13].

Alfa estrogen receptor (ERα), located on chromosome 6 is a protein comprising 595 aminoacids, with a molecular weight of 66 kDa. Therefore, similar to other hormone receptors, the estrogen receptor is a modulated protein that can be divided into six different domains (A-B-C-D-E-F) with specific functions [7,14].

Recently, a second receptor named beta (ERβ) was discovered. It is homologous to ERα and located on chromosome 14, comprising 485 aminoacids with a molecular weight of 54.2 kDa. Estrogen receptors α and β have homologous proteins in different domains [7,14].

Estrogen exerts a regulating effect on progesterone receptor, which is synthesized in response to estrogen action. The presence of progesterone receptor directly reflects activation and gene transcription of estrogen receptor, and is useful for evaluating the prognosis of tumor cells [15].

Human progesterone receptor gene is located on chromosome 11q22-23. Several different types of isoforms, particularly progesterone receptor A and B, have been well characterized and studied [16].

Progesterone receptor B is longer with 933 aminoacids, having a molecular weight of 114 kDa and five domains (A, B, C, D and E). Progesterone receptor A is shorter with 769 aminoacids, having a molecular weight of 94 kDa and also five domains (A, B, C, D and E) [17].

Other progesterone receptor variants are cited, including C, S and T [18,19].

Isoforms A and B of the progesterone receptor are functionally different and responsible for diverse physiological effects on different target tissues. However, these effects are not yet fully understood [17].

Ki-67 is a widely used antibody which reacts with a nuclear nonhistone protein (antigen). Ki-67 antigen is revealed as a double band with apparent molecular weights of 395 and 345 KD and is regulated by a gene located on chromosome 10 [21-26].

To evaluate the use of low-dose tamoxifen, we proposed this clinical trial administering tamoxifen at a dose of 10 mg/day for 14 days in the neoadjuvant treatment of invasive breast carcinoma.

## Patients and methods

### Patients

A prospective randomized clinical trial of 38 patients with invasive breast carcinoma was conducted at the Outpatient Facility of the Division of Breast Diseases, HMI, Goiânia, Brazil, and Division of Breast Diseases of the Federal University of São Paulo (UNIFESP), São Paulo, Brazil.

Women with palpable and operable breast tumors, stages I, II and IIIA participated in the study. Diagnosis had been confirmed by clinical exam, mammography and cytology positive for breast cancer.

Excluded from the study were women with tumors eligible for chemotherapy; neoadjuvant endocrine therapy; history of thromboembolism and breast cancer in the pregnancy-puerperal cycle; and previous biopsy of the breast lesion.

Patients were distributed into two research groups according to the randomized study. Group A, with 20 patients (control) and Group B, with 18 patients (tamoxifen 10 mg/day)

Patients in Group B (tamoxifen 10 mg) were advised to start medication on the day biopsy was performed for diagnostic confirmation, and continue for 14 days until the date of definitive surgery. Tamoxifen was administered at a dose of 10 mg once a day, always at the same time.

Similarly, Group A (control) patients underwent diagnostic biopsy. After 14 days, these patients returned for definitive surgery.

At the time of incisional biopsy, a sample of the tumor was fixed in 10% formalin and paraffin-embedded for diagnostic confirmation, as recommended by the World Health Organization. Another fragment was fixed separately in buffered formalin for less than 24 hours. During definitive surgery (mastectomy or quadrantectomy), a new tumor fragment was removed contralateral to the first sample collected, to avoid areas of necrosis. This fragment was fixed in buffered formalin. The remaining material was fixed in 10% formalin prior to paraffin processing and routine evaluation.

### Histopathological method

The breast tissue obtained was fixed in 10% buffered formalin for a maximum period of 24 hours prior to routine paraffin processing. Specimens were serially trimmed into slices of approximately three to four micrometers and mounted on slides stained by the hematoxylin-eosin technique for histopathology study.

The 10% buffered formalin solution was prepared by adding 100 ml formaldehyde (in a 35–40% solution) p.a., 4 g monohydrated sodium phosphate monobasic (NaH2P04.H2O), 6 g sodium phosphate dibasic anhydrous p.a (Na2HP04) and 1000 ml distilled water q.s.p.

### Immunohistochemical method

Tissue blocks of 4 to 5 μm thickness were cut with a microtome and prepared on silanized slides.

Tissue sections were deparaffinized with hot xylol at 60°C for 15 minutes at room temperature during more than 15 minutes. Histologic sections were then hydrated in decreasing concentrations of alcohol (100, 95, 80 and 70%) for 30 seconds each and rinsed in distilled tap water.

Antigen retrieval was then mediated by humid heat (water bath FANEN Mod. BM – 147, series RX 4614), submerging the slides in a buffer sodium citrate solution 10 mMol, pH 6.0, during five minutes [27].

Subsequently, the slides were cooled at room temperature and rinsed in tap water. Endogeneous peroxide block was performed with 0.3% oxygenated water (diluted in methanol) in three baths of five minutes each. Then slides were rinsed in distilled tap water and in PBS buffer (buffered saline solution with 0.01 M and pH 7.4 to 7.6).

Incubation with primary monoclonal antibody Ki-67 was then performed (clone MIB-1) DAKO, code N° M7240, batch 012, diluted to 1/150, at 4°C overnight, followed by rinsing in PBS solution.

Bacteria were then incubated with monoclonal antibody specific for estrogen receptor α, DAKO clone 1D5, code N° M7047, batch 070, diluted to 1:250.

Another batch of bacteria was sequentially incubated with monoclonal antibody specific for progesterone receptor, DAKO clone PgR 636, code N° M3569, batch 532, diluted to 1:650.

The next step was to visualize the reaction with chromogen substrate 3-3' diaminobenzidine DAB (0.06 g DAB and 1 ml H_2_O_2 _20 volumes) for three to five minutes at 37°C, completely covered to protect from light. On microscopy, a dark brown precipitation was observed, after rinsing under distilled tap water.

Sections were counterstained with Harris hematoxylin for one minute. The slides were then immersed in ammoniacal water and rinsed under distilled tap water.

Subsequently, the slides were dehydrated using alcohol (at 70, 80, 95 and 100% concentrations) three times for one minute each and diaphanized in three xylol baths for one minute each. Slides and coverslips were mounted with balsam (Entellan^®^).

### Quantitative study

The immune reaction of Ki-67 (MIB-1), estrogen receptor (1D5) and progesterone receptor (PgR 636) was quantitatively evaluated by counting a minimum of 1.000 cells [19]. The percentage of immune reaction in each case was obtained from the relationship between stained and unstained cells multiplied by 100. Only the epithelial tumor element of the sample was evaluated. The stromal element was excluded.

For the study, an image digital analysis system was used, consisting of a NIKON JAPAN LABOPHOT-2 microscope, series n° 440021, with a NIKON objective lens of 40×, attached to a color digital video camera SDC-312ND POWER: DC12 V 3 W SAMSUNG TECHWIN CO LTD series N° R4602662, to import images into an AUTHENTIC AMD microcomputer with an AMD DURON processor (tm) and 128.0 MB RAM and 40 GB HD; equipped with a digitized plate, with software WinTV32 and Windows Paint in Windows 98 Immune reaction was evaluated by an objective lens of 40× (final magnification of 400×) in 15 to 20 fields per case, categorizing as positive the cell with a typical brown nucleus.

### Statistical analysis

Analysis of variance was employed (ANOVA) to compare quantitative variables (age, menarche, tumor size and histologic grade) and evaluate homogeneity between Groups A (control) and B (tamoxifen 10 mg/day) [28,29].

A Kruskal-Wallis non-parametric test was used to evaluate homogeneity between Groups A and B, regarding variables (number of pregnancies, deliveries, abortions, classification by BI-RADS System, quadrant location of tumor, number of positive lymph nodes, total number of axillary lymph nodes and clinical staging) [30].

Fisher's exact test was employed to evaluate homogeneity between Groups A and B, regarding variables such as previous family history of breast cancer in first- degree relative, menopausal status, laterality and type of surgery [28].

A comparison between both Groups A and B regarding Ki-67 (MIB-1), estrogen receptor (ID5) and progesterone receptor positivity (PgR 636) before treatment was analyzed by the Mann-Whitney test [30].

The Wilcoxon non-parametric test was used to compare equality in mean expression of Ki-67 (MIB-1), estrogen receptor (1D5) and progesterone receptor (PgR 636) of Groups A and B, before and after tamoxifen therapy [30,31].

The level of significance was set at 5% (α<5%) in all tests and significant values were marked with an asterisk.

## Results

**Figure 4:**
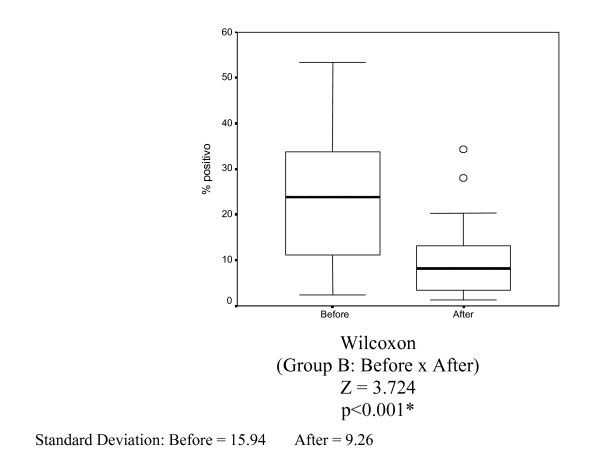
Box-plots for mean Ki-67 variables (MIB-1) in Group B (tamoxifen 10 mg) before and 14 days of treatment.

The mean percentage of nuclei stained by Ki-67 (MIB-1) in Group B (10 mg of tamoxifen) before and after tamoxifen treatment was 24.69% and 10.43%, respectively. Reduction was statistically significant (p < 0.001) (Table 4 and Figure 4).

**Table 4:** Percentage of nuclei stained by Ki-67 (MIB-1) in Group B (tamoxifen 10 mg) before and after 14 days of drug use (magnified 400×).

PATIENT	Before tamoxifen 10 mg (% stained cells)	14 days after tamoxifen 10 mg (% stained cells)
1	8.82	1.70
2	33.79	10.54
3	6.51	5.52
4	29.07	12.62
5	53.32	28.06
6	17.58	8.77
7	12.48	1.27
8	21.05	13.15
9	24.87	2.86
10	11.22	7.95
11	29.45	16.25
12	45.90	6.80
13	24.25	8.29
14	43.56	20.32
15	5.35	3.45
16	2.26	1.75
17	51.40	34.30
18	23.50	4.10

**MEAN**	24.69	10.43

**Figure 5:**
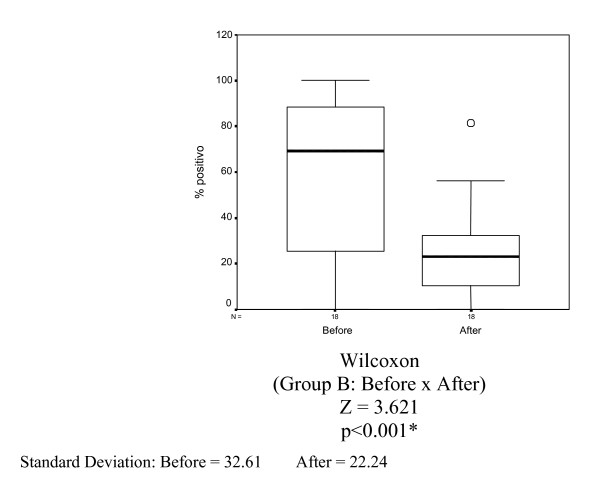
Box-plots for mean estrogen receptor variable (1D5) in Group B (tamoxifen 10 mg) before and after 14 days of treatment.

The mean percentage of nuclei stained by estrogen receptor (clone 1D5) in Group B (10 mg tamoxifen) before and after tamoxifen treatment was 59.53% and 25.99%, respectively. A statistically significant reduction was observed (p < 0.001) (Table 5 and Figure 5).

**Table 5:** Percentage of nuclei stained by estrogen receptor (1D5) in Group B (tamoxifen 10 mg) before and after 14 days of drug use (magnified 400×).

PATIENT	Before tamoxifen 10 mg (% stained cells)	14 days after tamoxifen 10 mg (% stained cells)
1	68.59	30.25
2	45.68	12.47
3	69.48	26.56
4	55.26	12.56
5	0.00	0.00
6	68.67	26.11
7	71.39	10.58
8	74.37	55.42
9	94.96	27.85
10	70.87	20.32
11	100.00	81.32
12	25.63	13.58
13	10.25	2.25
14	92.54	32.38
15	21.21	4.29
16	100.00	50.26
17	14.15	5.58
18	88.59	56.21

**MEAN**	59.53	25.99

**Figure 6:**
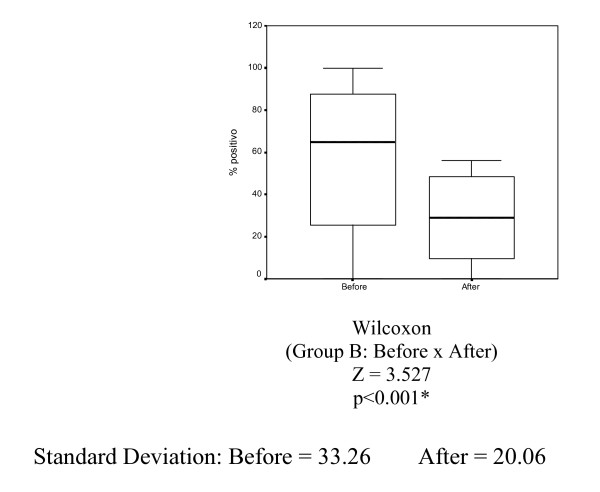
Box-plots for the mean progesterone receptor variable (PgR 636) in Group B (tamoxifen 10 mg) before and after treatment for 14 days.

The mean percentage of nuclei stained by progesterone receptor (PgR 636) in Group B (tamoxifen 10 mg) before and after tamoxifen treatment was 59.34% and 29.59%, respectively. Reduction was statistically significant (p < 0.001) (Table 6 and Figure 6).

**Table 6:** Percentage of nuclei stained by progesterone receptor (PgR 636) in Group B (tamoxifen 10 mg) before and after 14 days of drug use (magnified 400×).

PATIENT	Before tamoxifen 10 mg (% stained cells)	14 days after tamoxifen 10 mg (% stained cells)
1	89.63	48.26
2	85.62	56.21
3	15.62	20.34
4	65.49	49.25
5	25.64	2.45
6	50.47	9.87
7	85.49	30.58
8	55.21	41.59
9	0.00	0.00
10	71.64	24.47
11	2.87	0.00
12	100.00	48.47
13	63.98	53.41
14	60.32	25.48
15	20.29	2.45
16	98.79	48.78
17	89.64	26.98
18	87.49	44.15

**MEAN**	59.34	29.59

**Figure 1:**
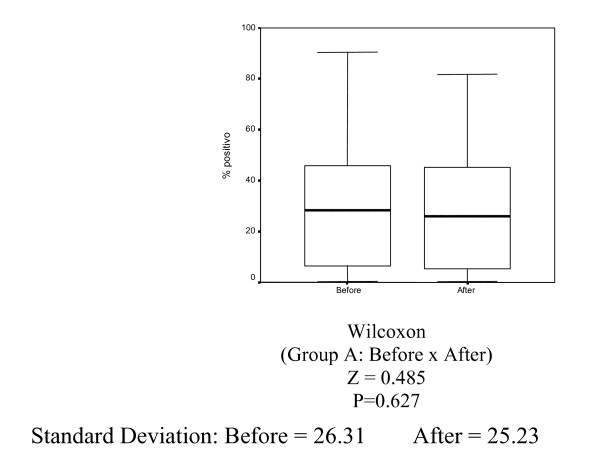
Box-plots for mean Ki-67 variable (MIB-1) in Group A (control) before and 14 days.

**Figure 2:**
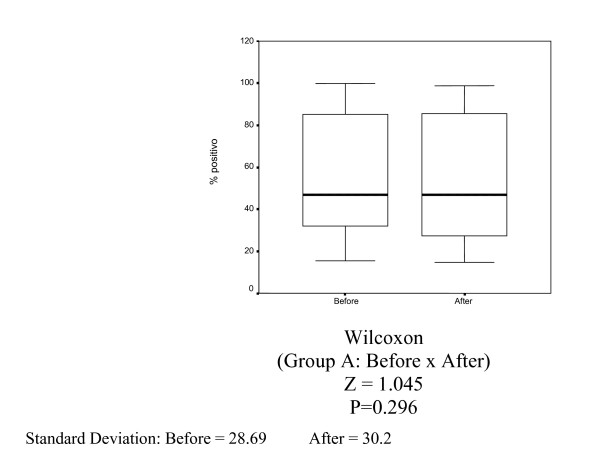
Box-plots for mean estrogen receptor variable (1D5) in Group A (control) before and after 14 days.

**Figure 3:**
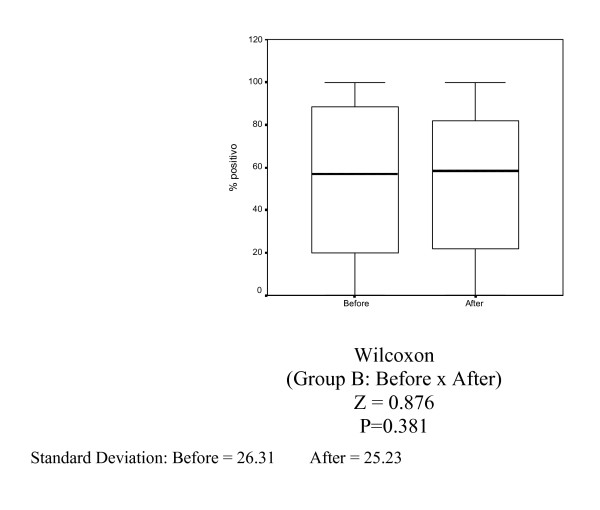
Box-plots for mean progesterone receptor variable (PgR 636) in Group A (control) before and after 14 days.

In Group A (control), there was no statistically significant decrease in Ki-67 (MIB-1) (p = 0.627), estrogen receptor (1D5) (p = 0.296) and progesterone receptor (PgR 636) positivity (p = 0.381) (Tables 1, 2 and 3, Figures 1, 2, 3).

**Table 1:** Percentage of nuclei stained by ki-67 (clone MIB-1) in Group A (control) before and after 14 days (magnification 400×).

PATIENT	Control (before) (% stained cells)	Control (after 14 days) (% stained cells)
1	29.31	26.12
2	30.83	31.24
3	75.35	69.56
4	28.17	5.68
5	28.39	25.30
6	42.60	40.51
7	20.32	25.63
8	49.25	50.01
9	50.39	52.18
10	70.21	68.92
11	21.52	30.61
12	10.52	5.23
13	2.14	5.76
14	1.23	1.52
15	90.54	81.87
16	2.36	3.49
17	0.25	0.36
18	32.56	36.48
19	2.56	4.89
20	12.62	8.98

**MEAN**	30.06	28.72

**Table 2:** Percentage of nuclei stained by estrogen receptor (1D5) in Group A (Control) before and after 14 days (magnified 400×).

PATIENT	Control (before) (% stained cells)	Control (after 14 days) (% stained cells)
1	45.61	33.82
2	46.31	33.45
3	25.63	30.48
4	47.41	44.82
5	60.63	55.96
6	33.89	48.78
7	88.92	90.81
8	33.59	29.51
9	30.25	14.65
10	15.47	25.26
11	30.12	22.23
12	100.00	97.01
13	81.12	80.58
14	51.25	53.69
15	16.05	20.15
16	95.87	98.79
17	80.56	75.89
18	45.06	22.79
19	89.07	90.46
20	98.19	98.89

**MEAN**	55.75	53.40

**Table 3:** Percentage of nuclei stained by progesterone receptor (PgR 636) in Group A (Control) before and after 14 days (magnified 400×).

PATIENT	Control (before) (% stained cells)	Control (after 14 days) (% stained cells)
1	49.92	26.45
2	63.51	70.84
3	33.56	25.98
4	98.71	90.29
5	85.59	78.36
6	66.89	71.24
7	78.42	77.25
8	50.14	45.89
9	0.00	0.00
10	16.52	14.81
11	0.00	0.00
12	100.00	98.89
13	95.12	96.48
14	91.24	78.25
15	17.85	19.25
16	80.13	85.64
17	25.36	22.84
18	15.12	20.98
19	22.05	26.97
20	100.00	100.00

**MEAN**	54.51	52.52

Both groups (A and B) were considered homogeneous before treatment, regarding Ki-67 (MIB-1) (p = 0.838), estrogen receptor (ID5) (p = 0.737) and progesterone receptor positivity (PgR 636) (p = 0.704).

Groups A and B were considered homogeneous regarding the following control variables: age (p = 0.481), age at menarche (p = 0.182), tumor size (p = 0.145) and histologic grade (p = 0.069), number of pregnancies (p = 1.000), deliveries (p = 0.857), abortions (p = 0.850), classification in the BI-RADS System (p = 0.544), quadrant location of tumor (p = 0.667), number of lymph nodes involved (p = 0.542), total number of dissected lymph nodes (p = 0.988), clinical staging (p = 0.368), previous family history of breast cancer in first-degree relative (p = 0.526), menopausal status (p = 0.159), laterality (p = 0.248) and type of surgery performed-mastectomy or quadrantectomy (p = 0.255).

In Group A (control), the usual infiltrating ductal carcinoma was the histologic variety in 100% of the cases, while in Group B (tamoxifen at 10 mg/day), infiltrating ductal carcinoma was predominant in 16 cases (90.0%). Only one case of mucinous carcinoma (5.0%) and one case of undifferentiated neuroendocrine carcinoma (5.0%) was observed.

## Discussion

Tamoxifen, a selective estrogen receptor modulator (SERM), is the standard drug for endocrine therapy in patients with estradiol and/or progesterone receptor positive breast cancer [1,4,35,38,39].

The response of estrogen-receptor positive tumors to hormonal therapy ranges from 30 to 60%, reaching 80% when there is concomitant positivity for both estrogen and progesterone receptors [34-36].

Interaction between growth factors and tamoxifen may lead to inhibition of cell proliferation in both receptor positive and receptor negative tumors. TGF-α inhibition and stimulation of TGF-β production is observed. In cell culture with estrogen positive receptors, TGF-β activation may occur through an autocrine loop. Nevertheless, in cell culture with estrogen negative receptors TGF-β might exert paracrine function, regulating proliferation adjacent to neoplastic cells [2,6,33,36,37].

Breast cancer is a hormone dependent malignancy. However, Beattie et al. (2006) observed that in the postmenopause there is no association between levels of estradiol, testosterone and SHBG (*Sex Hormone-Binding Globulin*) in patients with breast cancer compared to healthy women. Measurements of these sex hormones should not be used to identify women at risk for treatment or chemoprevention with tamoxifen [40].

In the past, tamoxifen was used in daily doses of 30 and 40 mg for treatment of advanced breast cancer. Subsequently, the dose was empirically reduced to 20 mg/day to decrease toxicity [2-4,37,41].

Uehara [42] evaluated estrogen and progesterone receptor positivity in invasive breast carcinoma in women receiving tamoxifen at a dose of 20 mg/day for two and 14 days. He observed a significant decrease in these receptors when tamoxifen was administered for 14 days. However, he found no significant difference when the drug was used for two days. These data suggest that 20 mg should be the empirical dose, since length of drug use was a greater determinant of decrease in tumoral proliferation than tamoxifen dose.

In a randomized, double-blind study, Decensi et al. [2] used tamoxifen in 120 women with hormone receptor positive breast cancer and observed a similar decrease in Ki-67 positivity with doses of 1, 5 and 20 mg/day for 28 days. In this study, the pharmacodynamic effect of these three different doses of tamoxifen was evaluated. A dose-response relationship was observed between several cardiovascular risk factors, such as concentration of total cholesterol, low density lipoprotein (LDL), insulinoid growth factor (IGF-1), fibrinogen, antithrombin III and SHBG. There was no substantial modulation of these risk factors. Results indicated that the conventional dose of 20 mg/day may be higher than the minimum dose necessary for the biological activity of tamoxifen. Lower doses would likely be sufficient to obtain an antiproliferative effect.

At the same time, Rodrigues de Lima et al. [4] observed a similar reduction in positivity of estrogen and progesterone receptors, Ki-67, apoptosis and mitotic index in normal breast epithelium with tamoxifen doses of 5, 10 or 20 mg/day.

In the following year, Kisanga et al. [41] evaluated Ki-67 variation after tamoxifen treatment during 28 days, using doses of 1, 5 and 20 mg/day. A significant reduction in Ki-67 was observed with the three different doses compared to the placebo group. There was a similar reduction using the three doses. Such evidence indicates that tamoxifen dose could be reduced without damage to its efficacy and with a decrease in side effects.

Such studies prompted us to set up this research project. Thus, a prospective, randomized clinical trial was carried out in breast cancer patients, using tamoxifen at a dose of 10 mg/day for 14 days. The study was simultaneously controlled by a group that did not receive drugs. The aim of the study was to evaluate effects specific to surgical intervention before and after 14 days of drug use, since complications including hematoma, inflammation, infection, use of anesthetics and other drugs are inherent in the procedure.

Randomization was adequate, because the control variables were uniform in both groups. There was no significant reduction in immunoreaction of the markers studied in the control group (without drug), showing that eventual complications from the surgical procedure did not hinder data analysis.

We emphasize that the microscope slides were examined and read twice by the research author. Control was supplied by a medical pathologist with proven experience in breast pathology. We also highlight that the three markers were studied for all 38 patients, six slides per patient, before and after treatment. For each slide, from 15 to 20 fields were selected counting at least 1000 cells, making 6.000 cells per patient.

The percentage of stained nuclei in Group B (tamoxifen – 10 mg/day) for the Ki-67 monoclonal antibody (MIB-1) before and after 14 days of drug use was 24.7% and 10.4%, respectively, showing a significant reduction.

The same occurred for the estrogen receptor, 59.5% before and 25.9% after drug use, and progesterone receptor, 59.34% and 29.6%, respectively. Both showed a significant reduction. Biomarker results suggest that lower tamoxifen doses produce results similar to those of the standard dose. If applied to clinical trials, such findings may lead to a significant reduction in costs and less side effects.

Our results are similar to those of Uehara [42], who also evaluated estrogen and progesterone receptor positivity in breast cancer of women treated with tamoxifen. However, tamoxifen was used at a 20 mg/day dose for 14 days. The mentioned author observed a ratio reduction from 55.4% to 10.2% in cells stained for estrogen receptors, and from 59.2% to 18.9% in those stained for progesterone receptors. In a similar manner, using a 10 mg/day dose of the drug for 14 days, our data revealed a reduction from 59.5% to 25.9% and from 59.3% to 29.6% for estrogen and progesterone receptors, respectively.

Likewise, results obtained by Decensi et al. [2] demonstrated that use of 1, 5 or 20 mg/day dose of tamoxifen for four weeks, lead to a similar reduction in proliferative activity of breast carcinoma, evaluated by Ki-67 monoclonal antibody. In the group using a tamoxifen dose of 1 mg/day, 4-hydroxytamoxifen metabolite level was 10–20 times higher than the minimum inhibitory concentration.

In the current study, the percentage of nuclei stained by Ki-67 monoclonal antibody after using 10 mg/day of tamoxifen for 14 days decreased from 24.7% to 10.4%. Consistent with this result, Descensi et al. [2] obtained a 21.2% to 14.0% decrease in nuclei staining, demonstrating that our results are similar to those obtained by the above-mentioned authors.

Our results showed that using 10 mg/day of tamoxifen for 14 days is enough to reduce proliferative activity. Descensi et al. [2] described that this activity was similarly reduced at doses of 1, 5 or 20 mg/day. The decrease in estrogen and progesterone receptor positivity in this study, at 10 mg/day of tamoxifen for 14 days was similar to that observed by Uehara [42] at 20 mg/day of tamoxifen for 14 days.

A limitation to the current study was not having used a group treated with 20 mg/day of tamoxifen. Future research should be undertaken to compare five randomized groups, using placebo or 1, 5, 10 and 20 mg of the drug.

The current study is a pilot project of a research line conducted in the Discipline of Breast Pathology at the Department of Gynecology of UNIFESP-EPM, apart from other studies that have already been published. It demonstrated efficacy *in vivo *of tamoxifen used at a low dose. From these data, we can propose studies into the effect of low-dose tamoxifen on the adjuvant and neoadjuvant therapy of breast cancer.

Although tamoxifen has been in clinical use for over 30 years in oncology, little is known about the minimum effective dose of the drug. This was probably due to the low number of adverse effects observed with the usual dose.

Recent studies employing aromatase inhibitors have demonstrated encouraging results with an improvement of disease-free survival, when compared to those using tamoxifen.

In the IMPACT (*Immediate Preoperative Anastrozole, Tamoxifen or Anastrozole Combined with Tamoxifen*) study, 292 women were randomized into three groups, anastrozol (n = 98), tamoxifen (n = 98), anastrozol combined with tamoxifen (n = 96) for neoadjuvant treatment of breast cancer. The authors observed a significant reduction in Ki-67 positivity, greater in anastrozol users than in tamoxifen users at a dose of 20 mg/day for 12 weeks. However, suppression of cell proliferation was similar in tamoxifen users versus users of tamoxifen combined with anastrozol. The authors observed an increase in disease-free survival in anastrozol users compared to tamoxifen users. No difference in disease-free survival was found in tamoxifen users versus users of tamoxifen combined with anastrozol [43].

Despite the introduction of a new generation of drugs that are ten times more expensive, tamoxifen still has a satisfactory cost/benefit ratio. The drug is also of major importance in countries with public health policies.

The researcher has responsibility for investing in research that may improve medical care of the low-income population, in spite of commercial interests. Dose reduction would be an important strategy for treatment in our setting, increasing the number of women who would have access to medication, with a minimum of side effects.

In addition to results obtained by other authors [2,3,41,42], our results support the need to conduct clinical trials on adjuvant therapy and chemoprevention with low-dose tamoxifen, to provide comprehensive and low cost medical care for women with breast cancer.
